# The challenge of institutionalised complicity: Researching the pharmaceutical industry in the era of impact and engagement

**DOI:** 10.1111/1467-9566.13536

**Published:** 2022-10-10

**Authors:** Paul A. Martin

**Affiliations:** ^1^ iHuman Institute University of Sheffield Sheffield UK; ^2^ Department of Sociological Studies University of Sheffield Sheffield UK

**Keywords:** careful engagement, complicity, orphan drugs, pharmaceutical industry

## Abstract

The pharmaceutical industry plays a central role in the production of the drugs we use to treat most illnesses. It is immensely powerful and has received sustained attention from sociologists of health and illness, who have provided a critique of its influence and sometimes unethical behaviour. However, in recent years, funders are increasingly expecting researchers to engage and collaborate with stakeholders, including industry. This raises important questions about the institutionalisation of complicity and the different forms this might take. This article asks: How can sociologists engage with the pharmaceutical industry in a positive and constructive manner, whilst remaining independent, principled and critical? It will draw on my experience of establishing a major project on high‐priced drugs for rare diseases and the literature on collaboration, stakeholder engagement and responsible research to propose a methodological framework to address this challenge. This is based on six *PRIMES:* (normative) Principles, Reflection and Independence, (field) Mapping, (careful) Engagement and Strategic intervention that have broad applications to many other areas of contemporary social science research.

## INTRODUCTION

The pharmaceutical industry plays a central role in the production of drugs we use to treat most illnesses. It is immensely influential, shaping understandings of the body and disease, as well as the development of health services and has received sustained attention from both the sociology of health and illness (SHI) and science and technology studies (STS) (Abraham, 2008; Busfield, 2021; Davis & Abraham, 2013; Dumit, 2012; Pollock, 2019; Sismondo & Greene, 2015; Williams et al., 2011). Within this body of work, there has been a wide range of topics and approaches. These include studies of drug development, regulation, marketing, finance, access to therapy and the impact on health care. However, with a few notable exceptions (e.g., Pollock, 2019), most of these studies have only involved limited interaction with industry, mainly via interviews with company executives or observations at conferences and during policy debates. In general, sociologists have chosen to maintain independence and a critical distance from directly working with Big Pharma. The resulting scholarship has highlighted a series of key issues, including regulatory failure and the adverse consequences of deregulation (Abraham, 2008; Abraham & Davis, 2020; Davis & Abraham, 2013); questionable marketing practices (Sismondo, 2018); the negative impact of financialisation (Busfield, 2020) and how industry shapes health‐care systems around private interests (Dumit, 2012). This body of work has contributed important evidence to policy debates and highlighted the sometimes unethical behaviour and major systemic problems associated with the activities of pharmaceutical companies.

In the last decade, there have been important shifts in the research system, with greater emphasis on ‘applied’ research and knowledge exchange. In the UK, this has inspired a series of major institutional changes and the rise of the ‘impact agenda’. One consequence of this is a much greater emphasis on social scientists working closely with stakeholders and the users of their research. It is increasingly difficult to get grants from some funders without having stakeholders on advisory boards or engaging them in a sustained fashion. There are significant benefits to this, including doing more well‐informed research, intervening directly in important commercial and policy debates. However, in this context, researchers who either are required or feel obliged by funders to engage or collaborate more closely with the pharmaceutical industry risk being accused of complicity and guilt by association. In this sense, complicity is becoming institutionalised. Working with this powerful actor raises major questions about research integrity, conflicts of interest and co‐option. This article will use a case study of a grant I was awarded in 2020 on high‐priced drugs for rare diseases to explore these issues. Although not formally requiring stakeholder engagement, in practice I believe this was a pre‐requisite to get the funding.

The notion of complicity is a loaded term and whilst it may be a productive concept in theorising the dilemmas of engagement with industry, it is not helpful in establishing working relationships given its inbuilt criticism. The paper will therefore ask: *How* *can sociologists engage with the pharmaceutical industry in a positive and constructive manner, whilst remaining independent, principled and critical*?

One of the first things to consider is that with Open Access publishing anything I write will be easily read by the groups and individuals I will be working with. In this sense, they are already looking over my shoulder and I’m conscious of their potential reaction even before we have established a working relationship. This demands both an ethic of care in fostering these fragile relationships and reflexivity to maintain personal and professional integrity. These new forms of entanglement will require a fresh vocabulary and novel forms of experimental practices with an acceptance that these may ultimately fail. This article therefore provides an opportunity to think through these issues. It is not based on empirical evidence but instead draws on my previous experience of research in this area and the existing literature. In undertaking this, I will use the concept of careful engagement to build a practical framework to minimise the risk of complicity. Such a framework is needed in the context of a changing research system that increasingly mandates stakeholder engagement and their involvement in the governance of social science research. Existing notions of good research practice are not fully equipped to navigate the dilemmas posed by this growing institutionalisation of complicity.

As a starting point, some important context will be given about the rise of the impact agenda and the consequences of this for research funding. This will be followed by an appraisal of both the positive contribution of the pharmaceutical industry and its more negative aspects as a way of defining possible areas of complicity. An analysis will then be made of the different social science standpoints taken when studying the pharmaceutical industry and concepts of engagement and collaboration. The research project on orphan drugs will be briefly described. The next sections will review sociological work on complicity, careful engagement and responsible research and innovation. Finally, these ideas will be used to develop a methodological framework for engagement that seeks to avoid complicity. This has a broad application to many other areas of contemporary social science research which engage with powerful actors.

## RECENT CHANGES TO THE RESEARCH SYSTEM AND THE ASSESSMENT OF GRANTS

There have been important changes in the research system over the last 20 years, with an increasing emphasis on links between academic research and its application in the real world as part of the move to a knowledge economy. This has been characterised as Mode 2 knowledge production and found institutional expression in the UK in several ways. Firstly, a new criterion of research impact was introduced in 2010 as part of the revised Research Excellence Framework (REF) used by the government to evaluate the quality of academic research (Williams & Grant, 2018). Impact is broadly defined as the contribution research makes outside the academy to society and the economy, and in the most recent (2020) REF impact constituted 25% of the overall score. In response the main funder of UK social science, the Economic and Social Research Council (ESRC), introduced impact as an important criterion in its grant assessment process. This was via a specific statement on ‘Pathways to Impact’ and more recently by including it as a goal throughout grant applications. This policy was implemented through the inclusion of expert user assessors on ESRC Grant Panels who ‘…include experienced people from across the public, business and civil society sectors to help ensure ESRC funds high quality research with academic, economic and societal impact’ (ESRC, 2021b). Although a focus on impact has not been adopted to the same extent by all research funders, this has helped shift the research culture making the achievement of external impact central to many of the UK’s social science projects.

Secondly, a series of major changes has taken place in the organisation of UK research funding. This saw the establishment of Innovate UK in 2007 to support business and research collaborations to accelerate innovation and has channelled a greater proportion of funding into applied research. In 2018, UK Research and Innovation (UKRI) was created by the merger of Innovate UK and the long‐established and more academically oriented research councils into a single unified structure that subordinated the ESRC to a broader mission of research excellence, collaboration and knowledge exchange to strengthen the UK economy. There is evidence that these policy and institutional changes have significantly altered the incentive structure in UK academia with greater emphasis and value attached to research impact (Grove, 2017; Parks et al., 2019). This growing pressure to engage with stakeholders, including powerful and dominant groups, risks institutionalising different forms of complicity.

These changing norms and expectations around impact have translated into greater emphasis on engaging and collaborating with potential users of social science research when designing projects. This was reflected in the feedback I received during the grant application process from staff in the Wellcome Trust, panel members and external reviewers. In particular, there was a strong steer to have a ‘balanced’ International Advisory Board that contained good representation from different parts of the pharmaceutical industry and other stakeholders. Letters of support from external stakeholders and users of research were also seen as an important way of demonstrating the value of the proposed research. Whilst neither of these were decisive in the ultimate funding decision which centred on the quality of the proposal, I was left with a strong feeling that having industry represented on the project advisory board was an essential pre‐requisite for getting funding.

Before outlining ideas for how to avoid complicity, it will be important to first make a balanced assessment of the role of the pharmaceutical industry in the creation of drugs for rare diseases. This will help identify both the potential benefits of engaging with companies, given their pivotal role in this area, as well as the complicities researchers might face.

## THE ROLE OF THE PHARMACEUTICAL INDUSTRY IN THE DEVELOPMENT OF NEW MEDICINES

Large multinational pharma companies (‘Big Pharma’) have come to dominate drug markets internationally. A more detailed assessment of their role, power and commitments is beyond the scope of this article but some overall features will be briefly noted. Firstly, the pharmaceutical industry has played the main role in developing the modern pharmacopoeia that exists in most health‐care systems. With the exception of some traditional forms of medicine (e.g., Chinese traditional medicine), the drugs we take to treat almost all health conditions have been manufactured by pharmaceutical companies. Many of these products are the result of large‐scale, sustained investment in research and development (R&D) amounting to billions of dollars every year. Secondly, drug discovery and development is a risky business with a high attrition rate and relatively few products gain significant sales. However, those drugs that are successful have very large ‘blockbuster’ sales of over $1 billion a year. Total global pharmaceutical sales were $1228 billion in 2020. As a consequence of these risks and rewards, financial markets expect a high return on investment (RoI), with the industry being one of the most profitable in the modern economy. Thirdly, the pharmaceutical industry is highly regulated by the government. This also serves as a major barrier to entry for smaller companies, and there is a close relationship between companies and governments in many countries. The pharmaceutical industry is one of the most influential and well‐funded political lobbies in the world.

However, there are important caveats to this. Firstly, the widely held view that it is only industry that discovers new drugs has been challenged by evidence that US National Institutes of Health funding contributed to the basic research associated with all of the 210 new drugs approved by the US FDA from 2010 to 2016 (Cleary et al., 2018). A more accurate model of the drug innovation system would therefore fully recognise the importance of the public sector. This was clearly demonstrated by the creation of COVID vaccines during the pandemic (e.g., Oxford University/AstraZeneca).

Secondly, the claims made about the very high costs of drug development, which underpin the justification for both high prices and RoI, have been disputed. A long held view in policy discussions is that it costs $1–2 billion to bring a drug to the market, but the real cost may be much lower. The not‐for‐profit drug developer, Drugs for Neglected Disease initiative (DNDi), estimates that the cost of developing a new chemical entity is $100–150 million (DNDi, 2014). Instead, high prices are often a function of pressure from investors.

Thirdly, there has been very significant criticism over decades of the unethical and illegal behaviour of some pharmaceutical companies, including:

- Secrecy, lack of transparency and suppression of negative clinical trial results (Goldacre, 2012).
- Exploiting participants in clinical research in developing countries (SOMO, 2008)
- Manipulating the regulatory system through capture, corporate bias and deregulation, resulting in reducing of some drug safety standards (Abraham & Davis, 2020; Davis & Abraham, 2013)
- Promoting drugs with limited additional benefits, for example, evidence that as many as 50% of new medicines in the EU do not represent any added therapeutic advance for patients (EPHA et al., 2014).
- Lack of investment for conditions that mainly affect people in low‐income countries (Wirtz et al., 2017)
- Mis‐selling, aggressive and unethical marketing (Skandrani & Sghaier, 2016). For example, the current prescription opioid crisis in the USA.
- Excessively high prices for some new medicines and massive increases (‘price gouging’) in generic drug prices in recent years (Busfield, 2021)

These criticisms are reflected in the fines imposed on the industry. For example, between 2003 and 2016, US Federal, State and Securities regulators imposed fines of over $33 billion for illegal activity on 22 of the top 26 pharmaceutical companies (Arnold et al., 2020). These included penalties for bribery, anti‐competitive practices, non‐disclosure, misleading and off‐label marketing and pricing violations. However, at the same time, it should be recognised that the industry, working in partnership with regulators, scientists and clinicians, has started to address a number of these issues (e.g., release of clinical trial data). Despite this, much more needs to be done.

Although the assessment of the role of the pharmaceutical industry in relation to drugs for rare diseases has many of the same overall features as outlined above, it also has a number that are distinct. In most cases, drugs are the only treatment option for these mainly genetic disorders. Over the last 20 years, there has been a rapid increase in the number of novel medicines for rare diseases that were previously untreatable. This had led to very significant improvement in outcomes for some conditions. However, there remain a number of problems, including the ultra‐high price of some new medicines and unequal patient access; lack of transparency in pricing; concerns about potential abuses of orphan drug incentives and the difficulty in evaluating and reimbursing new cell and gene therapies.

Having outlined these problems, we can now better answer what complicity might look like. This would include lending legitimacy, failing to call out or question unacceptable practices; avoiding difficult or controversial topics; ignoring the views and experience of patients and other citizens, especially marginalised groups; taking too narrow a view of the problems to be studied or failing to consider alternative options or futures. The next section will develop a conceptual framework to help address these issues.

## DIFFERENT SOCIAL SCIENCE STANDPOINTS ON RESEARCHING THE PHARMACEUTICAL INDUSTRY

As mentioned above, there is a wide range of different approaches to research on the pharmaceutical industry. With respect to a discussion of complicity, these vary both in terms of their normative stance and the level of methodological engagement with companies. On the one hand, there is work that is strongly *committed* to particular normative or political values (e.g., objections to profit in health care) and is generally critical of the pharmaceutical industry. This includes research done in collaboration with patients as well as scholarly activism with campaign and advocacy groups.

In contrast some social sciences play a more *enabling* role working directly with companies. Here, research may be clearly in the service of industry to improve products and expand markets or aims to work with a range of stakeholders including firms to promote shared goals. A good example of this is academic health economic assessment of new medicines. In some cases, these are sponsored by companies to help obtain reimbursement. The UK ESRC promotes engagement with business and has several schemes to foster academic–industry collaboration.

In between these two positions is work that seeks to maintain an *agnostic* normative starting point. Although much research in SHI and STS may be characterised as ‘critical’, this is not to say that the normative starting point is already fixed or committed. Instead, they are evidence‐based critical inquiries into the activities of the industry, how it is governed and the implications this has for society, health and health care.

In addition to these different normative positions, there are also various modes of social science knowledge production. These have traditionally been defined according to the data sources and methods used (e.g., quantitative vs. qualitative). More recently there has been growing interest in different forms of engagement and collaborative knowledge production. Here, I have tried to define three distinct but overlapping methodological approaches:

1) *Independent*—in which the researcher keeps a distance from industry or other powerful groups. This may involve surveys, qualitative interviews, attending industry events and dialogue with critics within the industry. However, the research does not generally involve active dialogue with mainstream representatives of companies on research findings and the formulation of policy proposals.
2) *Engaged*—this is similar to the independent mode in the techniques used to collect data but may also involve a greater level of ethnographic work within the pharmaceutical sector, collecting in depth data on industry and other stakeholder perspectives. Here, the aim is to include data that might not be gathered by a strictly independent approach. However, what makes this approach distinct is the direct engagement with stakeholders through their membership of advisory boards, interactive events and active dialog. This represents an important shift in the governance of research with greater oversight of social science research practices, findings and recommendations by other actors. Whilst this does not necessarily end independent judgement and could lead to more robust research findings, it opens up a complex micro‐politics in which social scientists may have to negotiate criticism of their work from stakeholders who disagree with their findings and interpretation of evidence.
3) *Collaborative*—this includes contract research, joint working and different forms of co‐production. The methods listed above might be used in addition to innovative co‐production techniques and joint research design and data analysis. This marks another shift in governance, with the surrender of independence and joint control over the core processes of knowledge production.

If we consider these two separate dimensions—normativity and methodological—a matrix of nine different epistemic standpoints can be produced:

1. *Critical analysis (committed/independent)*—Where the social scientist adopts a strong (and critical) normative position but works independently from any stakeholder.
2. *Advocacy (committed/engaged)*—The research is carried out with the explicit aim of assisting service users or marginalised groups and is engaged with them.
3. *Academic activism (committed/collaborative)*—Social scientists working jointly with campaign and advocacy groups to support their goals.
4. *Detached (agnostic/independent)*—Traditional role of the detached and independent academic in an ‘ivory tower’.
5. *Interactive (agnostic/engaged)—*Research that is actively engaged with a range of stakeholders but seeks to remain impartial and separate from any of them.
6. *Consensus building (agnostic/collaborative)—*Social scientists working closely with a range of stakeholders to build a common position on topics of mutual interest.
7. *Consultancy (enabling/independent)—*Research commissioned by industry, often to work on areas of commercial relevance.
8. *Stakeholder consultation (enabling/engaged)—*Commercially focused research that directly engages patients and other stakeholders.
9. *Participatory co‐design (enabling/collaborative—*Co‐production projects involving collaboration between industry and other stakeholders.

These different positions are summarised in Table 1 and illustrated using examples from the broad field of social science research on pharmaceuticals.

**TABLE 1:** Examples of the different standpoints in social science health research

Level of engagement/Normative position	Independent	Engaged	Collaborative
Committed	*Critical analysis*: Research exposing the corrupting influence of pharmaceutical marketing on biomedical knowledge production (Sismondo, 2013)	*Advocacy*: Partnership of patients, clinicians and carers setting priorities for research in multiple sclerosis (MS Society, 2014)	*Academic activism*: Critical report on pharmaceutical innovation and access to medicines written with campaign and advocacy groups as co‐researchers (IIPP, 2018)
Agnostic	*Detached*: Analysis of drug discovery and development systems (Hopkins et al., 2007)	*Interactive*: Multi‐stakeholder study of value and cost of oncology drugs (Latimer et al., 2021).	*Consensus building*: Project using Delphi methods and consensus conference to develop policy on ensuring compliance in the use of medicines (EC, 2012)
Enabling	*Consultancy*: Academic study of patient access to medicine commissioned by the pharmaceutical company MSD (Kamphuis et al., 2021)	*Stakeholder consultation*: Research on the benefits of engaging patients in the drug development process (Stergiopoulos et al., 2020)	*Participatory co‐design*: User input into the design of pharmaceutical packaging (Lorenzini et al., 2017).

This analysis highlights differences in the approaches and standpoints that may be taken during research but also how knowledge production is governed. This has important implications for the problem of complicity. What I have described as *enabling* social science is inherently at risk of being complicit. In contrast, more *committed* approaches avoid this by largely not engaging with the mainstream pharmaceutical industry in any direct fashion.

As greater levels of engagement with Big Pharma are being institutionalised by funders, this inevitably involves occupying a more ambiguous and sometimes contradictory position that is fraught with difficulty. I have labelled this standpoint in Table 1 as ‘*interactive*’, and the later section of the paper will outline an approach based on the notion of careful engagement for how this might be achieved whilst avoiding complicity. Before doing this, more details of the case study project and how it might become complicit are outlined.

## THE RESEARCH PROJECT AND ENGAGEMENT WITH PHARMACEUTICAL COMPANIES

In February 2020, I was granted a £1M Wellcome Trust Investigator award to study high‐priced orphan drugs in the USA and the UK in a project titled ‘Orphan drugs: high prices, access to medicines and the transformation of biopharmaceutical innovation’ which started in early 2021. A brief description of the background to the project is given in Appendix A. The main focus of this research is on the development of and access to medicines for rare diseases, termed orphan drugs, following the introduction of the 1983 US Orphan Drug Act. Conceptually, the project will develop ‘orphanisation’ as a new theoretical tool to examine the extent to which the technologies, business models and high prices associated with the increasing production of orphan drugs are contributing to a major transformation of the contemporary pharmaceutical sector. In this sense, orphanisation has multiple dimensions, including changes in the structure of the pharmaceutical sector; processes of drug innovation; price and value of medicines; patient access to therapy; regulation, evaluation and reimbursement and the relationship between patient organisations (POs) and industry. These operate at multiple macro, meso and micro levels and together constitute a sociotechnical system where change in one domain coevolves and shapes change in other domains. Here, actors are guided by a series of shared beliefs, norms, expectations, routines, regulations and institutionalised practices which constitute a sociotechnical ‘regime’. Theory building will draw on previous work on pharmaceuticalisation (Williams et al., 2011) and studies of sociotechnical systems in health care (Boon et al., 2008) to further develop this idea. The aims of the project are shown in Box 1.

BOX 1 **Aims of project as stated in the application**

1 **Research questions: *To what extent is orphanisation occurring in the UK and USA? How is it being shaped by different technologies, institutions and actors? What are the implications for industry, health policies and patients?***

**Aims:**

1. Chart the growing industrial development of orphan drugs in the UK/EU/USA and analyse the emergence of new orphan business strategies.
2. Understand the role of different actors in the evolution of orphan drug policy, map the controversy around high prices and analyse proposals to improve access and reform orphan incentives.
3. Describe the crisis in pharmaceutical innovation, and explore the emergence of new forms of patient engagement in drug development.
4. Assess the implications of orphanisation for health and health care and develop proposals for more sustainable models of pharmaceutical innovation.

In addition, the project also has an implicit normative agenda concerning the responsible governance of innovation, the price of drugs, patient access to therapy and how we might better organise important aspects of drug discovery and development. These are the areas where the project may have a meaningful policy impact but also where the dangers of complicity lie.

### Engaging with stakeholders

The research will involve a wide range of qualitative and quantitative methods to collect data, including the analysis of policy documents, regulations and materials produced by companies; the analysis of regulatory information and proprietary industry databases; interviews with company managers, patient groups, policymakers and regulators; social media analysis including network mapping and ethnographic observation at industry events. As mentioned above, stakeholder engagement will take place via an International Advisory Board (IAB), which includes representatives from major pharmaceutical companies, rare disease patient organisations and regulatory agencies. The IAB will be consulted during the course of the research to gain their input and feedback on agenda setting, initial findings, policy proposals and dissemination. The project will also run a series of stakeholder workshops in its later stages to get critical feedback on findings and help craft proposals for change. These will involve the use of scenarios for future development, use and governance of orphan drugs based on the initial findings. The project will also experiment with creative arts‐based interactive methods to get the input of patients.

Whilst meaningful engagement has significant advantages, it raises the possibility that some of the research activities and results may be uncomfortable for different stakeholders. It is of course hoped that research conducted with integrity will be widely accepted as valid knowledge, but this cannot be guaranteed if findings touch on sensitive policy issues that might threaten commercial or other interests. This could lead to disputes with and between some stakeholders and different forms of complicity. These might include the research team’s self‐censorship to avoid conflict. More importantly, the need to ensure stakeholder engagement and avoid criticism may have a chilling influence on the research agenda. In the worst case, the research team may be complicit by adopting the norms and values of dominant actors and lose both independence and criticality. How might these risks be minimised?

## DEVELOPING A CONCEPTUAL FRAMEWORK TO THINK ABOUT ENGAGEMENT, COLLABORATION AND RESPONSIBILITY

### Forms of (social science) complicity

One response to the growth of social science engagement and collaboration with external partners has been the provision of guidance by funders and professional associations (e.g., ESRC, 2021a; US SSRC, 2018) and the development of a series of ethical frameworks for the conduct of research (AoIR, 2019; EC, 2018). However, whilst such guidelines may be useful, they often do not consider possible complicities.

There are a small number of social science papers that have explored the challenges and pitfalls of working with powerful or dominant groups and which highlight different forms of complicity. A useful starting point for thinking about this is the dilemma posed by Donna Haraway in asking how can we both recognise our complicity in damaging and violent practices while also working to change them? (2016, p. 35). Within the field of biomedicine, a group of social scientists have explored the tensions in working with STEM researchers, industry and policymakers around the development of synthetic biology. This has involved an attempt to move beyond the ethical, legal and social implications (ELSIs) of controversial research and towards a ‘post‐ELSI’ space of collaboration. This requires a commitment to experimentation, taking risks, collaborative reflexivity, opening‐up discussions of unshared goals and neighbourliness (Balmer et al., 2016). However, the experience of Claire Marris and Jane Calvert during their participation in the drafting of the UK Roadmap for Synthetic Biology highlights the difficulties in doing this (Marris & Calvert, 2020). Whilst they were invited to participate in the Committee that drew up the Roadmap, they felt powerless to change embedded assumptions about the future trajectory of the technology or open up broader societal debates. They did succeed in including a commitment to responsible innovation within the Roadmap but ended up questioning how seriously this would be taken and ultimately felt ‘implicated, and even complicit’ (p. 55) in making a policy they did not support.

In the domain of security, Evans et al. (2020) have explored the idea of ‘critical collaboration’ between social scientists and security communities of practice (e.g., defence analysts and military staff). Key challenges to doing this include, on the one hand, being too distant from the community resulting in accusations of being co‐opted by ‘the enemy’ (i.e., critics of security services), and on the other hand, being too close and becoming co‐opted and reproducing dominant discourses. Undertaking such critical collaboration involved developing a shared language and taking risks in raising unpopular topics. Despite these challenges, in some instances they felt able to follow a post‐ELSI approach as defined above (Evans et al., 2020, p. 17). In measuring the benefits of engagement, they adopted a processual understanding of success focused on collaborative forms of action, reflection and resulting shared responsibility (p. 18).

Within multidisciplinary studies of addiction, there has been a debate about the dilemmas posed by accepting funding from alcohol and pharmaceutical companies that manufacture addictive products (Miller et al., 2017). Many researchers have experienced problems in terms of restrictions placed on the research design, the publishing and sharing of data and attempts to undermine the policy impact of such studies. One approach to addressing these dilemmas is the use of decision‐making frameworks. An example of this is the PERIL framework proposed by Adams (2007), which looks at multiple criteria and provides a structured means of evaluating individual situations from an ethical perspective.

### Careful engagement

Another way of trying to navigate these dilemmas has been proposed by Lydahl and Nickelsen who call for ‘developing the understanding of “careful engagements” as a generative mode of knowledge production that take place between researchers and their research fields’. (Lydahl & Nickelsen, 2021). In developing this idea, they draw on several approaches in STS. Zuiderent‐Jerak (2015) proposes that research engagement may be seen as a form of intervention or situated experiments that are themselves generative of new knowledge. The other key idea is that of care and caring as elaborated by Puig de la Bellacasa (2017). This raises questions about what we want to care for, what we ourselves care about and the worlds our research and engagement is helping construct. In particular, Lydahl and Nickelsen propose the idea of ‘critical care’ as an approach that highlights questions of power and the exclusions produced and reproduced through caring practices. They ask ‘What inequalities do we risk producing especially when there is funding involved in the research? … How does careful engagement affect academic work and output?’ (Lydahl & Nickelsen, 2021, p. 3).

Engagement can also be seen as performative in creating certain associations within a field. Bruun Jensen (2007) refers to this as sorting attachments in which the researcher works out how to engage with other actors, institutions or agendas as part of conducting research (p. 239). No engagement is innocent as all actors come with existing cultural, political and economic relationships as well as institutionally sanctioned commitments (Bruun Jensen, 2007, p. 239).

In becoming carefully engaged, the researcher may choose to form certain attachments as a means of bringing in other voices, values and commitments to help shape the research agenda. At the same time, the researcher may become enrolled and be complicit by giving legitimacy to the activities and agendas of other actors. Avoiding this will require careful problem framing, balanced discussions and reflexive work to identify the issues and normative agendas important to the various actors involved.

### Responsible research and innovation

Responsible research and innovation (RRI) is an alternative approach to working with external stakeholders and aims to align the processes and outcomes of innovation with societal values and priorities by involving a wide range of stakeholders from a very early stage. Whilst there are different formulations of RRI, they generally share a commitment to a set of fundamental principles (Stilgoe et al., 2013). These have been most clearly articulated in the AREA framework adopted by research funders such as the UK Engineering and Physical Sciences Council. This continuously seeks to *Anticipate* impacts, *Reflect* on the purposes and potential implications of the research, *Engage* in an inclusive way and *Act* to influence the direction and trajectory of research and innovation (EPSRC, 2021).

There is now a burgeoning literature on many aspects of RRI. One important development is the idea of societal alignment, which aims to engage multiple and often diverse stakeholders, frame societal needs and align the goals and processes of science and innovation to meet these needs (Ribeiro et al., 2018).

In the health care and biomedical field, the principles and practices of RRI have been evaluated and developed further (e.g., Demers‐Payette et al., 2016) and several limitations have been identified, including the need to define the dimensions of responsibility and consider important aspects of systemic change. However, there is almost no literature within this tradition that applies RRI principles to working with the pharmaceutical industry. In the next section, a framework will be proposed that draws on the ideas outlined above for how to do this whilst minimising the risks of complicity.

## A FRAMEWORK FOR ENGAGEMENT WITH THE PHARMACEUTICAL INDUSTRY

This article does not aim to create new methods for working with the pharmaceutical industry, as there are plenty of well‐established tools for doing this. Instead, it seeks to develop a methodological framework based on *(normative) Principles, Reflection and Independence, (field) Mapping, (careful) Engagement and Strategic intervention (PRIMES)* to explicitly address the risk of complicity. Whilst the implicit notion of good research practice provides a useful starting point, it is not sufficient to address the novel challenges posed by the increasing institutionalisation of stakeholder engagement. A more explicit and formally structured approach is needed for this task.

### (Normative) principles

The idea of complicity is profoundly normative, and to avoid the risks outlined above, it is really important for the research project to adopt a well‐defined set of norms—both in terms of process and overall aims—against which the extent of complicity can be judged. As mentioned above, there are multiple ethical codes that have been proposed for the conduct of collaborative social science research and several well‐established ethical frameworks for the overall normative aims of the project. These concern access to health services and medicines in line with the commitment of the World Health Organisation (WHO) Sustainable Development Goals, the WHO Roadmap for Access to Medicines (WHO, 2019b) and the World Health Assembly resolutions on transparency and access to medicines (WHO, 2019a). They have the advantage of being already negotiated with broad international support and several major pharmaceutical companies have issued clear policy commitments to promoting sustainable and equitable access to medicines (e.g., AstraZeneca). A key task will be to translate these broad principles into a more concrete set of research objectives for any given project (see Box 2).

BOX 2 **Research objectives at strategic sites of investigation and engagement**

1 **Objective 1: Analyse the main barriers and possible solutions to ensure timely and equal access to effective rare disease therapies**

A range of financial and institutional barriers prevent equal access to orphan drugs, these include high prices, restrictive reimbursement and delays in technology assessment. A series of policy and regulatory changes are being introduced to improve access.

- Key sites of investigation:
  ○ Controversies and policy debates around barriers to equal access to orphan drugs in both market‐based (USA) and managed (UK) health‐care systems.
  ○ The development of novel forms of evidence and assessment that better capture rare disease patient experience.
  ○ Alternative access schemes that promote early/conditional approval of novel orphan therapies.

**Objective 2: Investigate how to establish the fair pricing of orphan medicines**

The fair price of a medicine is one that is affordable for health systems and patients whilst providing sufficient market incentive for industry (WHO, 2021). A range of stakeholders have called for greater price transparency and a clearer link between price, patient benefit and the investment made by companies. If very expensive therapies are to be widely adopted, sustainable payment models will need to be developed.

- Key sites of investigation:
  ○ Proposals for fair pricing and price transparency of orphan products.
  ○ The reimbursement of very expensive gene and cell therapies that may require long‐term monitoring.

**Objective 3: Explore the creation of more sustainable innovation systems for new orphan drugs**

There is a pressing need to increase the number of orphan products reaching the clinic and reduce development times and costs. This may need the creation of a more mixed economy of drug innovation.

- Key sites of investigation:
  ○ Current trends in the development, approval and price of orphan drugs and the implications of this for health‐care systems.
  ○ New models of Open Innovation that seek to reduce the cost and increase the speed of orphan development.
  ○ Alternative financial mechanisms for supporting orphan drug development and proposals to reform relevant legislation.

**Objective 4: Examine patient engagement in policy debates on access to high‐priced treatment and the development of new orphan medicines.**

There is growing interest in different forms of patient engagement in the development of new medicines for rare diseases. Patient groups are also being involved in trial design, decentralised data collection and health technology assessment.

- Key sites of investigation:
  ○ Patient involvement in the assessment and regulation of orphan drugs.
  ○ New models of patient group led drug development.
  ○ The experience of patients involved in industry trials for expensive gene and cell‐based therapies.

### Reflexivity and independence

Throughout the project, an ongoing reflexive approach will be adopted. This involves thinking about the experience and practices of undertaking the research to enhance objectivity and understanding of how our own actions, perspectives and values influence knowledge production, so that they can be changed where necessary (Bolton, 2010). This will be important in ensuring that an independent standpoint is maintained as a means of avoiding the complicities outlined above. A reflexive approach is multidimensional and involves both formal and informal processes. Its starting point is the normative principles, aims and objectives described above. Other forms of reflexive practice might include:

- Regular discussions within the research team
- Annual review with the Advisory Board
- Running workshops to develop the idea of careful and critical engagement with other social scientists who might act as ‘critical friends’
- Organising panels at international conferences to explore a wider range of experiences in engaging with the industry

### Mapping the field

At the start of the project, it is valuable to carefully map the sociotechnical landscape to be studied and to understand the power dynamics operating in it. As the research progresses, a better knowledge of this makes it easier to focus on key issues of concern and strategic sites of investigation and engagement. This can be achieved through background scoping as well as immersion in the field. A series of questions can guide this:

- *Actors and commitments*—Who are the key players? What forms of power and material and cultural resources do they control? What inequalities exist between actors? What commitments are they pursuing?
- *Institutions and regimes*—What are the organisations and institutions that define the sociotechnical regime?
- *Politics and alliances*—What issues are the focus of political contestation? What forms of politics are being pursued? Where do political alliances exist?
- *Trajectories*—What are the path dependencies in technology, policy and governance that result from the alignment of actors, commitments and institutions?

As the mapping progresses, it will also become possible to understand how the main actors and the interactions between them animate a particular domain. A key element shaping their behaviour is the sociotechnical imaginaries associated and embedded in the field (Jasanoff, 2015, p. 25). The analysis of sociotechnical imaginaries is similar to the call in RRI to anticipate and can be used to open up discussions during engagements and look at how they play an important part in political dynamics by helping to coordinate joint action.

Field mapping may sound like extra work, but it should be stressed that this is not the case. Researchers often do this as a tacit process during many social science research projects when building internal models of the world they study. Making this more explicit can help understand our entanglement in networks of power and how to better engage with different stakeholders.

### Careful and critical engagement

It is important to consider who to involve when engaging, and given the commitment to promoting inclusion and health equity, there is a need to look beyond the ‘usual suspects’ to ask who is currently excluded, silenced or marginalised. For example, groups traditionally excluded from clinical trials. However, there are very real limits to who can be easily included in some controversial discussions, with critical academics and NGOs being unacceptable to industry in a way that would make having them in the same room impossible. This highlights the way powerful actors avoid scrutiny on contentious issues through strategies of either direct suppression of debate or deliberate non engagement. However, creative methods can be used to ensure a full range of perspectives are considered even in the absence of a formal dialogue. On the other hand, the presence of pharmaceutical companies in interactive events may undermine the confidence of some groups in both their ability to fully participate and the integrity of the research if the research team is seen as too close to the industry. It should also be stressed that the pharmaceutical industry is not monolithic, with generic manufacturers, small biotechs, service providers and technology developers behaving quite distinctly from larger integrated companies. Capturing this variety is important.

There are multiple ways in which researchers can engage with the actors in their field of study. These include well‐established approaches such as qualitative interviews, observation at conferences and debates in policy forums. However, as outlined above, what makes the ‘interactive mode’ of knowledge production distinct from a more independent one is the involvement of stakeholders in dialogue and the governance of research. The two main ways this will be done in the orphan drug project are through a stakeholder membership of the Advisory Boards and participation in interactive project workshops. Advisory Board members will be asked to comment on project design, help articulate common problems, enable access and give feedback on findings, papers and policy recommendations. At an individual level, they will give specialist advice on specific aspects of the sector. The Board will therefore provide a space for collective discussion with the research team around the main issues of concern. Similarly, the interactive stakeholder workshops will focus on key topics in the later stages of the research. Here, draft findings, recommendations, scenarios and alternative futures will be scrutinised and debated with the aim of making outputs more robust. Careful and critical engagement in these processes will depend on several factors. Firstly, good interpersonal relations based on care, integrity and trust built up through an emphasis on openness and transparency. Secondly, carefully sorting attachments to give a balanced input from a range of perspectives to ensure no single actor dominates proceedings. Thirdly, ensuring the issues being researched and discussed are carefully chosen and framed so that multiple societal interests are aligned around strategic issues and common problems.

### Strategic sites of investigation and engagement

As mentioned above, an important part of the framework is to translate the project’s broad aims into a narrower set of normative objectives. These objectives are essential to avoid complicity and should be tightly focused on the main problems in any particular area; in this case, they are the challenges associated with ensuring affordable, timely and equal access to effective medicines.

In formulating such objectives, the research team will have to walk a line between making sure the most important issues of concern are addressed whilst doing this in a way that will enable engagement. In most fields of sociotechnical change, there are specific issues that multiple groups agree are key sites of contestation and controversy around which significant elements of a regime may be renegotiated. The outcome of the struggles will be important in determining the future direction of a field. They therefore represent *strategic sites of investigation and engagement* and speak to the call in RRI to influence the direction of innovation.

A detailed list of normative objectives can thus be developed in relation to these sites and used to form a concrete research agenda. A key consideration is the extent to which the shared commitments of different actors may align around these sites. This opens up the possibility of productive joint work. Adopting a post‐ELSI approach, the role of the social scientist in this context is to promote dialogue, create evidence, challenge assumptions and develop alternatives. A worked example of this is given in Box 2 in relation to this project on orphan drugs.

This list includes issues in which industry, patients, regulators and health policymakers all have a stake and can form the basis for careful engagement around a clear research agenda that avoids complicity.

## CONCLUSION

The starting point for this article was the question: *How can social scientists engage with the pharmaceutical industry in a positive and constructive manner, whilst remaining independent, principled and critical*? In addressing this, I have used the idea of (institutionalised) complicity to outline the potential dangers inherent in engagement and drawn on different bodies of empirical and conceptual work to develop a methodological framework for thinking through these issues. This is based on *(normative) Principles, Reflection and Independence, (field) Mapping, (careful) Engagement and Strategic intervention (PRIMES)*. It is hoped that this will help social scientists to work more successfully with the industry and guide future research in this area.

This article also contributes to academic debates about post‐ELSI collaboration, careful engagement and RRI. Firstly, it highlights the way in which the promotion of engagement and impact by funders institutionalises various forms of complicity. Secondly, it makes the case for an explicitly normative set of principles that anchor the research, communicate the values of the research team to all participants and can be used to reflect on the risks of complicity. Thirdly, it draws on theories of sociotechnical system change to stress the need for strategic mapping of the research domain as a means of analysing the key actors, their resources and power as well as their commitments and alliances. This will help identify participants, sort the research team’s attachments to different actors and facilitate reflexivity by better locating ourselves in the wider web of normative commitments. Fourthly, it places the notion of a regime of shared beliefs, norms, expectations, routines, regulations and institutionalised practices at the centre of analysis. This is useful in helping identify strategic sites of investigation and engagement around which it may be possible to successfully interact with a range of actors, including the industry. As a worked example, the paper also establishes a detailed agenda for research on the challenge of getting access to effective medicines for rare diseases.

However, from a critical perspective, the promotion of the impact agenda and the mandate to engage with powerful stakeholders, regardless of their commitments and track record, is a form of restriction or compulsion. This threatens the independence of the social sciences. In making research the servant of economic prosperity through the promotion of knowledge exchange, the scope for criticism is radically curtailed by both changing norms in the research community but also new forms of oversight and governance associated with stakeholder engagement. In this sense, it may be useful to think about how complicity is being institutionalised into research projects. This is not just a problem for pharmaceutical studies but is relevant to many other areas, including social science projects on mining, energy production, financial services, military and security services, farming and the use of animals in research etc. Whilst the proposed methodological framework seeks to raise awareness of this risk, only reasserting the benefits of truly independent research that can speak truth to power will in the long run protect the integrity of our work.

## AUTHOR CONTRIBUTION

Paul A. Martin is the sole contributor for this article.

## Data Availability

Data sharing is not applicable to this article as no new data were created or analysed in this study.

## APPENDIX A RESEARCHING HIGH‐PRICED DRUGS FOR RARE DISEASES

Historically, there has been a lack of treatment for the vast majority of the ~7000 rare genetic diseases, with only a handful available as recently as the 1990s. This was largely the result of a lack of investment from the industry, as many of these conditions only affect tiny patient populations and are therefore commercially unattractive. As a means of addressing this market failure, legislation—the Orphan Drug Act (ODA), 1983—was introduced in the USA.

Orphan drugs are defined by the ODA as products for conditions affecting <200,000 people. This provided incentives (e.g., tax breaks and extended market exclusivity) to stimulate new treatments for neglected patient groups. Similar policies have since been introduced in many other countries and have proved highly successful. The number of orphan drugs has increased rapidly with >500 US products by 2020 and sales growth of 12% p.a., projected to reach $240 billion (~20% of global drug sales) by 2024 (Evaluate Pharma, 2019). The combination of limited competition and small markets has led to orphan drugs being very expensive; the average cost of the top 100 US orphan drugs was $150,900 p.a. compared to $33,600 for the top 100 non‐orphan products in 2018 (Evaluate Pharma, 2019). A series of gene therapies that provide a ‘one‐off’ cure for some rare genetic diseases have recently been licenced, with some costing over $1M. As a result, orphan drugs are very profitable and account for a significant proportion of all therapies in company product pipelines. They have attracted massive investment and stimulated new business models based on high priced products for niche markets. This has prompted claims that it represents a major industrial transformation—so called ‘orphanisation’ (Vogler et al., 2018)—where common diseases are broken into small genome‐defined sub‐types which qualify for orphan status. However, the extent of orphanisation is disputed.

One of the most important issues in contemporary health care is the increasing cost of medicines. This is most apparent in the USA where these are not controlled by the government and the price of commonly used speciality branded drugs increased by 57% between 2014 and 2018 (Kamal et al., 2019). The very high price of orphan drugs has provoked a growing concern in many countries that patients are being denied access to life‐saving treatment due to these costs. A major debate on how best to improve patient access is ongoing. Important questions include the role of orphan drug incentives in supporting high prices and the lack of any transparent relationship between patient benefit and price. A number of initiatives have been taken to improve access, including new ways of evaluating products (e.g., use of Real World Evidence), accelerated access schemes and payment by results (e.g., value‐based pricing). The growing cost to health‐care systems of expensive orphan products risks undermining established norms in the allocation of resources and increasing inequality.

This has given new impetus to criticism of the established pharmaceutical innovation system as ‘broken’ and characterised by low productivity, market failure and therapies with little clinical benefit (IIPP, 2018). A series of initiatives are being taken to tackle these issues, including greater emphasis on open innovation and growing numbers of patient organisations (POs) are becoming actively involved in drug development.

Within the social sciences relatively little work has focused explicitly on orphan drugs (Douglas, 2015; Rabeharisoa & Doganova, 2016). The most relevant scholarship is on the increasing role of rare disease patient activism in science and politics. A series of mainly European case studies have explored the role of POs in collaborative scientific research (Pinto et al., 2017), political claims (Edwards et al., 2014) and policy development (Rabeharisoa & O’Donovan, 2014). These have often been conceptualised as forms of ‘evidence‐based activism’.
